# Plasma osteopontin levels in patients with head and neck cancer and cervix cancer are critically dependent on the choice of ELISA system

**DOI:** 10.1186/1471-2407-6-207

**Published:** 2006-08-15

**Authors:** Dirk Vordermark, Harun M Said, Astrid Katzer, Thomas Kuhnt, Gabriele Hänsgen, Jürgen Dunst, Michael Flentje, Matthias Bache

**Affiliations:** 1 Dept. of Radiation Oncology, University of Würzburg, Germany; 2 Dept. of Radiation Oncology, Martin Luther University of Halle, Germany; 3 Dept. of Radiation Oncology, University of Lübeck, Germany

## Abstract

**Background:**

The tumor-associated glycoprotein osteopontin (OPN) is discussed as a plasma surrogate marker of tumor hypoxia and as an indicator of the presence of pleural mesothelioma in asbestos-exposed individuals. The clinical introduction of plasma OPN measurements requires the availability of a reliable enzyme-linked immunosorbence assay (ELISA).

**Methods:**

We compared previously described and currently available ELISA systems on 88 archival plasma samples obtained from patients with head and neck or cervix cancer between 20 days before and 171 after the start of radiotherapy.

**Results:**

Median (range) plasma OPN levels were 667 (148.8–2095) ng/ml and 9.8 (3.5–189.5) ng/ml for a previously described and a newly marketed assay, respectively. Although results for different assays were significantly correlated (r = 0.38, p < 0.05, Spearman rank test), between-assay factors ranged from 2.0 to 217.9 (median 74.6) in individual patients. OPN levels in cervix cancer patients were comparable to those of head and neck cancer patients.

**Conclusion:**

Commercially available OPN ELISA systems produce different absolute plasma OPN levels, compromising a comparison of individual patient data with published results. However, different assays appear to have a similar capacity to rank patients according to plasma OPN level. A review of literature data suggests that plasma OPN levels measured even with identical ELISA systems can only be compared with caution.

## Background

Recent publications have renewed interest in the measurement of OPN, a tumor-associated glycoprotein secreted into bodily fluids, in the plasma of tumor patients. Plasma OPN level was shown by Le et al. to correspond with Eppendorf electrode measurements of tumor oxygenation in patients with head and neck cancer [1], suggesting a role for OPN as an endogenous marker of tumor hypoxia. In a landmark study, Overgaard et al. showed that only patients with high plasma levels of OPN (upper tertile) significantly benefitted from the addition of nimorazole, a hypoxic radiosensitizer, compared to standard radiotherapy in patients with head and neck cancer [2]. OPN may therefore serve as a marker by which to select head and neck cancer patients for intensified, hypoxia-specific, treatment. It is currently unclear whether OPN may be similarly useful to predict tumor hypoxia in other entities in which oxygenation also has prognostic impact such as cervix cancer [3,4]. Additional interest in OPN was raised by a prominent publication showing that in individuals with asbestos exposure, serum OPN levels can distinguish between those without cancer and those with pleural mesothelioma [5]. A molecular mechanism for the intracellular accumulation of OPN under hypoxia has recently been described [6,7], although secretion of OPN may require additional steps [8,9].

A widespread application of OPN measurement in patient plasma or serum is dependent on the availability of reliable commercial enzyme-linked immunosorbent assay (ELISA) systems and may be affected by the problem of comparability between results of different assay systems. The choice of material, e. g. plasma vs. serum, and duration of storage may also affect the results of the ELISA.

To the authors' knowledge, the ELISA system utilized in the two reports on plasma OPN as a marker of tumor hypoxia in head and neck cancer [1,2], is no longer commercially available We therefore performed a comparative analysis, using different commercial ELISA systems, of plasma OPN levels in 88 archival samples obtained from patients with head and neck cancer or cervix cancer at different timepoints before, during or after radiotherapy. Data were compared to published results for cancer and non-cancer patients as well as normal controls obtained with these ELISA systems.

## Methods

Eighty-eight archival citrate plasma samples obtained from patients treated with radiotherapy between December of 1998 and May of 2000 at the Martin Luther University of Halle, Germany, and stored at -80°C, were now analyzed. Of these, 34 samples were from eleven patients with head and neck cancer (ten males, one female) and 54 were from 12 patients with cervix cancer. The median number of samples per patient was 4 (range 1 to 7). In head and neck cancer patients, a median of 2 samples (range 1 to 7) and in cervix cancer a median of 5.5 (range 1 to 7) samples were available. Samples were collected between 20 days before and 171 days after initiation of radiotherapy. Plasma sample collection was performed after written informed consent and approved by the local ethics committee

Plasma samples were analyzed in triplicate with different commercial ELISA systems, according to the respective manufacturers' instructions, as follows. Aliquots of all samples were analyzed with the currently available TiterZyme ELISA kit (Assay Designs, Ann Arbor, MI) introduced in July of 2005 by this manufacturer ("assay A"). Aliquots of all cervix cancer samples were additionally analyzed with the ELISA system previously marketed by this manufacturer under the same name which was used in previous publications on OPN as an endogenous hypoxia marker [1,2]. This older version of the TiterZyme assay was designated "assay B1". According to this manufacturer, this assay (old TiterZyme, B1) was supplied by a third party producer (IBL, Gunma, Japan) who still markets this assay. All head and neck cancer samples were additionally analyzed with the current IBL assay which should be identical with the old TiterZyme assay and was therefore designated "assay B2". In summary, each sample was measured with one newly introduced ELISA assay (A) and one older assay (B) marketed under two different names (B1 and B2).

For each sample and assay, the mean of the triplicate measurement was calculated. The factor of the difference between assay B and assay A was determined for each sample. The correlation of results obtained with the newer assay (A) and the older assay (B) was analyzed by calculation of the Spearman-rank correlation coefficient (p < 0.05 regarded as significant).

## Results

**Figure 1:**
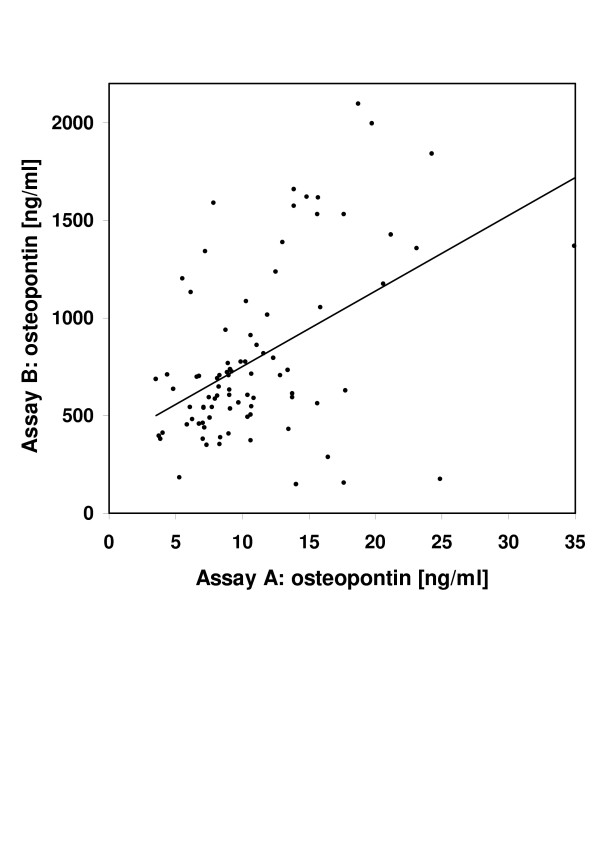
Osteopontin levels in individual plasma samples obtained from cervix or head and neck cancer patients undergoing radiotherapy. All samples were measured with two different ELISA systems, a newly introduced assay (A) and an older assay (B) marketed under two different names (see text). The results of both assays were significantly correlated (p < 0.05, Spearman rank test). For clarity, two data points with values of 80.5 and 189.5 ng/ml for assay A are not displayed.

The plasma OPN levels determined with assay A and assay B are displayed for the overall group and separately for patients with cervix cancer and head and neck cancer, respectively, in Table 1. In the overall set of samples, results obtained with assay A, the newly introduced assay, were consistently much lower than those obtained with assay B, the one frequently used in published reports. The median and mean factors by which the results of the two assays varied in individual patients were 77.1 and 74.6, with a range between 2.0 and 217.9. The results obtained with assay A and B were significantly correlated, as shown in Fig. 1 (r = 0.37, p < 0.05). The two versions of assay B marketed by different companies (B1 used in cervix and B2 used in head and neck cancer samples), although not compared against each other in identical samples, produced similar results in comparison with assay A (median factor of 70.3 for B1 in cervix and of 76.5 for B2 in head and neck), confirming that B1 and B2 can be regarded as identical.

Although the time points at which samples were taken were not necessarily comparable between cervix and head and neck cancer patients, both assays indicated similar plasma OPN levels in these two tumor entities with median OPN levels (head and neck vs. cervix) of 10.4 vs. 9.5 ng/ml (assay A) and 650.2 vs 667.0 ng/ml (assay B).

## Discussion

OPN has been studied as a blood tumor marker since the mid-1990. Its expression level has been linked to the presence or extent of metastases or to clinical progression in several tumor entitities including breast, prostate and lung cancer [review in [10]]. Most of the earlier studies were performed with individually developed ELISA systems preventing comparison of results between centers.

OPN has recently received renewed interest as a surrogate marker of tumor hypoxia in head and neck cancer, a disease entity in which oxygenation is of prognostic importance [11]. The association of plasma OPN levels with tumor oxygenation in head and neck cancer patients [1], their association with prognosis after radiotherapy and the benefit from a hypoxia-specific additional treatment (nimorazole) only in patients with high OPN levels [2] suggested that plasma OPN may in fact be a clinically useful surrogate marker of tumor oxygenation. These interesting results are a motivation to measure plasma OPN prospectively in future clinical trials of treatments targeted at hypoxic tumor cells or retrospectively in stored plasma samples from other relevant patient groups and compare the results to plasma OPN levels reported for head and neck cancer patients. However, the ELISA assay kit (TiterZyme by Assay Designs) used in the reports on plasma OPN in head and neck cancer [1,2] was recently replaced by a different assay marketed under the same name. According to information from this manufacturer, the older assay is still marketed by a different company (IBL). In published reports on plasma OPN, both older assays (Assay Designs old and IBL), which should yield identical results, have been used (Table 2) but so far no results have been published for the new Assay Designs ELISA system to the authors' knowledge.

We therefore compared these different ELISA systems on a set of 88 archival plasma samples from patients treated with radiotherapy for head and neck or cervix cancer. The main finding was that the OPN levels determined with the new assay were much lower – by a mean factor of 77 – than measured with either form of the older assay. There was no uniform factor between old and new assay that would permit calculation of one from the other. In individual samples the factor ranged from 2 to 218. Nevertheless, OPN levels determined with the older and the newer assay were significantly correlated, suggesting that both assays should have a similar potential for selection of patients with poor-prognosis, hypoxic tumors. The fact that both forms of the older assay had similar mean factors compared to the new assay supports equivalence of the two. The large difference between old and new assay is well explained by differences in the epitope recognition sites of the respective capture antibodies provided with the ELISA assay kits and is, at least in part, anticipated by the range of standards provided with the respective kits (5–320 vs. 2–32 ng/ml). Various molecular forms of OPN have been described, resulting from RNA splicing, glycosylation, phosphorylation and proteolytic fragmentation, e. g. by thrombin [12]. The use of various combinations of antibodies raised against four different portions of human OPN in a total of six separate ELISA systems has been shown to recognize distinct truncated or glycosylated forms of OPN [13].

A review of published data for the two forms of the old assay reveals that mean plasma OPN levels for healthy volunteers, for instance, vary substantially even when the identical ELISA kit is used (Table 2). This may in part be explained by the different type of material analyzed, such as EDTA plasma vs. citrate plasma vs. heparin plasma. Some authors did not specify the type of plasma obtained for OPN detection or analyzed serum samples, which apparently results in lower levels of OPN in healthy volunteers (Table 2). Conditions and duration of storage may also be critical. As highlighted by the literature review, many common non-malignant conditions, including coronary artery disease, interstitial pneumonia and other benign pulmonary diseases, multiple sclerosis or liver dysfunction may cause elevated plasma OPN values [14-17]. Some of these conditions may be present to a varying degree in groups of "healthy" volunteers, depending also on the age group selected.

## Conclusion

As a consequence of the present results, the plasma OPN levels obtained with commercially available ELISA assay kits must be interpreted with caution. Differently optimized kits may be marketed under the same name such that comparison with published results may be compromised. It appears that even despite the use of the identical ELISA system, OPN levels are not reproducible in comparable patient cohorts between laboratories. For instance, the median values for untreated patients with advanced head and neck cancer were 450 ng/ml and 113 ng/ml in the two previously published series [1,2]. However, elevated plasma OPN levels were consistently reported for different groups of cancer patients compared to controls, regardless of the type of assay used. For any multi-center investigation involving the measurement of plasma OPN, the collection and storage of plasma should be strictly regulated and the ELISA analysis should be centralized.

## Competing interests

The author(s) declare that they have no competing interests.

## Authors' contributions

DV designed the study, performed statistical analysis and drafted the manuscript.

HMS analyzed plasma samples and reviewed the manuscript.

AK analyzed plasma samples and reviewed the manuscript.

TK treated the patients, collected plasma samples and reviewed the manuscript.

GH treated the patients, collected plasma samples and reviewed the manuscript.

JD treated the patients, collected plasma samples and reviewed the manuscript.

MF performed statistical analysis and reviewed the manuscript.

MB designed the study, collected plasma samples, performed statistical analysis and drafted the manuscript.

All authors read and approved the final manuscript.

## Pre-publication history

The pre-publication history for this paper can be accessed here:



## Figures and Tables

**Table 1:** Plasma osteopontin levels in head and neck cancer and cervix cancer patients as measured using two different ELISA systems.

**patient group**		**assay A**	**assay B**	**individual factors (assay B/A)**
		osteopontin (ng/ml)	osteopontin (ng/ml)	
**all samples **	*mean ± SD*	13.9 ± 21.0	786.0 ± 442.5	77.1 ± 41.4
**(n = 88)**	*median (range)*	9.8 (3.5–189.5)	667.0 (148.8–2095.0)	74.6 (2.0–217.9)
				
**head and neck cancer**	*mean ± SD*	16.1 ± 31.1	794.3 ± 467.2	76.3 ± 38.9
** (n = 34)**	*median (range)*	10.4 (3.8–189.5)	650.2 (148.8–1841.0)	76.5 (5.7–184.5)
				
**cervix cancer**	*mean ± SD*	12.4 ± 11.0	780.7 ± 430.6	77.6 ± 43.3
** (n = 54)**	*median (range)*	9.5 (3.5–80.5)	667.0 (155.5–2095.0)	70.3 (2.0–217.9)

**Table 2:** Overview of osteopontin levels measured with commercially available ELISA systems in healthy controls, malignant and benign diseases.

**assay**	**material**	**patient group (n)**	**osteopontin mean ± SD (ng/ml)**	**osteopontin median (range) (ng/ml)**	**ref.**
**Assay Designs (B1)**	citrate plasma	head and neck cancer (54) von Hippel Lindau disease (31) healthy controls (15)	447 (261–843) 318 (233–461)	450	[1]
	plasma	head and neck cancer (320)	--	113 (12–1382)	[2]
	plasma	nasopharyngeal cancer healthy controls	184.6 (6.8–856.9) 75.9 (10–455)	-- --	[18]
	EDTA plasma	multiple sclerosis – relapsing patients (30) healthy controls (10)	-- --	280 174	[17]
	serum	ovarian cancer (51) healthy controls (28)	49 11	-- --	[19]
					
**IBL (B2)**	serum	pleural mesothelioma (76) asbestos-related non-malignant disease (69) healthy controls (45)	133 ± 10 30 ± 3 20 ± 4	-- -- --	[5]
	EDTA plasma	esophageal carcinoma (103)	--	606 (82.8–1980)	[20]
	plasma	ovarian cancer (51) healthy controls (107)	486.5 147.1	-- --	[21]
	plasma	ovarian cancer (38)	--	178 (12–3468)	[22]
	serum	ovarian cancer, post-surgery (267) healthy controls (67)	74 ± 116 147 ± 192	25 (25–671) 85 (25–617)	[23]
	heparin plasma	non-small lung cancer (158) benign pulmonary disease (54) healthy controls (25)	356.2 175.6 29.5	319.1 (16–1600) 161.6 (1.3–442.8) 17.9 (0–102.8)	[24]
	EDTA plasma	coronary artery disease (107) healthy controls (71)	616 ± 308 443 ± 237	-- --	[16]
	EDTA plasma	interstitial pneumonia (17) healthy controls (20)	945 ± 256 156.7 ± 52.2	-- --	[15]
